# Quantification of the Anti-diabetic Effect of Allium cepa

**DOI:** 10.7759/cureus.59174

**Published:** 2024-04-27

**Authors:** Amba Esakki, Ramya Ramadoss, Lakshmi Ananthapadmanabhan, Sandhya Sundar, Suganya Panneerselvam, Pratibha Ramani

**Affiliations:** 1 Dentistry, Saveetha Dental College and Hospitals, Chennai, IND; 2 Oral Pathology and Oral Biology, Saveetha Dental College and Hospitals, Chennai, IND

**Keywords:** blood glucose level, alpha-glucosidase inhibition, alpha-amylase inhibition, onion peel, ethanolic extract, anti-diabetic effect

## Abstract

Background: Allium cepa, or onion, boosts numerous health benefits, including anti-diabetic effects. Its rich array of antioxidants and sulfur compounds not only aids heart health by lowering cholesterol and blood pressure but also exhibits anti-inflammatory properties. Onion's antibacterial and antiviral properties help combat infections, while its compounds like quercetin show promise in cancer prevention. Additionally, Allium cepa supports respiratory health by relieving coughs and colds and aids digestion with its prebiotic properties. Incorporating onions into a balanced diet can enhance overall well-being, including managing blood sugar levels in individuals with diabetes.

Aim and objective: This study aims to determine if the ethanolic extract from the dried peel of Allium cepa holds potential as an anti-diabetic agent, with a focus on its ability to manage diabetes and reduce blood sugar levels.

Methodology: To prepare the ethanolic extract from dried onion peel, the peel was finely ground and soaked in ethanol. The mixture was then agitated and filtered to separate the liquid extract. Finally, the filtrate was concentrated using methods such as rotary evaporation or vacuum distillation to obtain a concentrated extract for further analysis like alpha-amylase inhibition assay and alpha-glucosidase inhibition assay.

Results: The ethanolic extracts derived from dried onion peel demonstrate inhibition of alpha-glucosidase, leading to reduced blood glucose levels. Additionally, this inhibition prompts an increase in insulin production.

Conclusion: The study underscores that the efficacy of the ethanolic extract of dried onion peel increases with concentration. It highlights the presence of beneficial compounds like total phenolics, flavonoids, quercetin, and its derivatives in onion peel, known for their therapeutic roles in cardiovascular health, weight management, diabetes control, cancer prevention, and antimicrobial activity. These findings affirm the hypoglycemic and anti-diabetic properties of Allium cepa's ethanolic leaf extract.

## Introduction

Diabetes mellitus, classified as a metabolic disorder, disrupts the metabolism of proteins, fats, and carbohydrates [1]. It encompasses various types such as Type 1, resulting from the destruction of pancreatic beta cells, and Type 2, associated with insulin resistance and reduced insulin production, alongside gestational diabetes during pregnancy and other forms. Type 2 diabetes, affecting 90-95% of cases, is characterized by progressive insulin resistance and beta-cell dysfunction [2]. This chronic hyperglycemia poses significant health risks, leading to microvascular and macrovascular complications such as retinopathy, nephropathy, neuropathy, cardiovascular diseases, and stroke. Treatment goals include preventing complications, regulating blood glucose levels, and managing hyperglycemia through lifestyle modifications and pharmacological interventions. Additionally, addressing other risk factors like hypertension, dyslipidemia, and obesity is crucial in improving overall quality of life for individuals living with diabetes [3].

Diabetes is characterized by impaired metabolism of carbohydrates, proteins, and fats due to insufficient insulin production, insulin resistance, or both. Persistent hyperglycemia, the hallmark symptom, is closely associated with the development of acute and chronic complications [4,5]. Key objectives in diabetes management include achieving glycemic control, understanding insulin regulation, and preventing associated complications. Oxidative stress, stemming from heightened external free radical production, is a primary factor contributing to diabetic complications [6,7].

Many types of drugs have been developed to control diabetes with remarkable results, despite the fact that a number of the generated goods have side effects because medicines for diabetes are lifelong [8,9]. Therefore, there is a rising interest in medicinal plant-based products in the diet of the general public in order to decrease the usage of medications and, consequently, their side effects, as well as to achieve the greatest possible level of preventative result [10,11]. The widely grown and eaten vegetable onion is a superb provider of both macro- and micronutrients [12].

Onion peel, often overlooked as biowaste alongside the bulb, harbors a plethora of phytochemicals. While conventional plant-based therapies for diabetes have been extensively studied, emerging research highlights the anti-diabetic, hypocholesterolemic, and antioxidant properties of onion peel extract and powder [13,14]. Incorporating biowaste onion peel extract into dietary supplements for targeted populations promises far-reaching benefits for the environment, economy, and health. With diabetes mellitus surging globally, the demand for nuanced and targeted therapies is escalating. Despite traditional remedies being utilized for generations, only a handful of plant-based treatments have undergone rigorous scientific evaluation for diabetes management [15,16].

In line with recent recommendations from the WHO Committee on Diabetes, emphasizing the exploration of traditional or ancient methods for managing type 2 diabetes mellitus, this study aims to investigate the potential benefits of increasing doses of Allium cepa (red onions), particularly the extract derived from onion peels. Considering economic constraints and the affordability and accessibility of herbal products, the study seeks to ascertain the potential consequences of escalating doses of red onion extract, aligning with the WHO's call for further analysis of traditional diabetes management approaches.

## Materials and methods

Preparation of ethanolic extract of dried onion peel 

**Figure 1:**
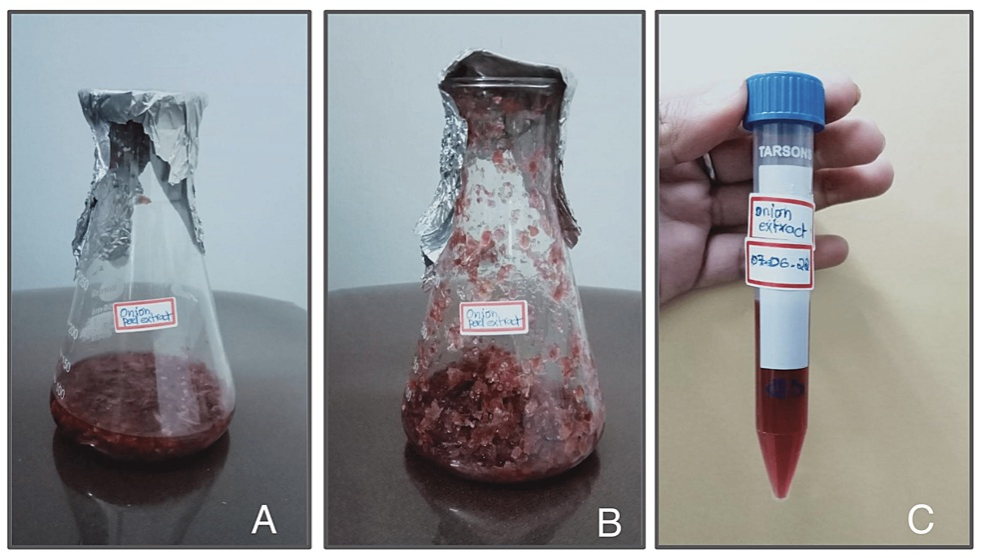
A - Dried onion peel in ethanolic extract, B - Boiled sample, C - Filtered and cooled extract

To prepare the ethanolic extract of dried red onion peel, the outer layer of the red onion was collected and dried. The dried peel was then finely powdered using a mixer grinder. Subsequently, 5g of the powdered onion peel was added to 50ml of ethanol, and the mixture was placed in a shaker for 48 hours to facilitate extraction. After extraction, the solution was boiled for five minutes using a heating mantle and allowed to cool. The cooled solution was then filtered using Whatman filter paper to remove any solid particles. The filtrate was further concentrated by boiling, and ethical clearance was obtained for the study (Figure 1). 

Alpha-amylase inhibition assay

One possible therapeutic approach for illnesses involving the intake of carbohydrates, like diabetes and obesity, is to inhibit the enzyme alpha-amylase, which is involved in the breakdown of starch and glycogen [17]. The inhibition of alpha-amylase was carried out. In short, to make a total volume of 500 microliters of reaction solution, 490, 470, and 450 L of buffer were added to a variety of quantity (10, 30, and 50 mL) of 30 mg/mL of onion extract and stored (at the room temperature at 37°C and 4°C) and calcined (300°C, 500°C, and 700°C). Subsequently, the reaction containers were filled with 500 microliters of alpha-amylase and 1,000 microliters of starch. After that, the process of reaction vessels were incubated for five minutes at 100°C in a water bath. This is followed by the addition of 500 microliters of NaOH. The percentage inhibition of a-amylase was calculated as ((Ao - Ai)/Ao)×100, where Ao was the absorbance of the standard and Ai was the absorbance of the test samples.

Alpha-glucosidase inhibition assay

Compared to alpha-amylase inhibitors, alpha-glucosidase inhibitors are a more effective class of anti-diabetic medications that can reduce hyperglycemia, particularly postprandial hyperglycemia [18]. When it comes to particular postprandial hyperglycemia, alpha-glucosidase inhibitors are a more effective category or group of antidiabetic medications than alpha-amylase inhibitors. After eating, there is a quick rise in blood glucose levels known as postprandial hyperglycemia. To ascertain the extract and fractions' alpha-glucosidase inhibitory activity, a few minor adjustments were made to the conventional protocol. A 96-well plate was used for the reaction mixture, which included 20 μl of extract and fractions at different concentrations (0.1, 0.2, 0.3, 0.4, and 0.5 mg/ml) that were pre-incubated for 15 minutes at 37°C, 50 μl of phosphate buffer (100 mM, pH = 6. 8), and 10 μl of alpha-glucosidase (1 U/ml). The mixture was further incubated for 20 minutes at 37°C. Each of these studies was run in triplicate, with the control condition being the same: ignoring the test material.

## Results

Alpha-amylase activity

The ethanolic extract derived from dried onion peel (*Alium cepa L*) has demonstrated promising anti-diabetic properties by inhibiting alpha-amylase activity. This inhibition leads to a reduction in blood glucose levels and a concomitant increase in insulin production. In our study, we compared the efficacy of this extract, obtained from Allium cepa, with that of a standard anti-diabetic drug, such as acarbose, which operates through a similar mechanism of alpha-amylase inhibition.

**Figure 2:**
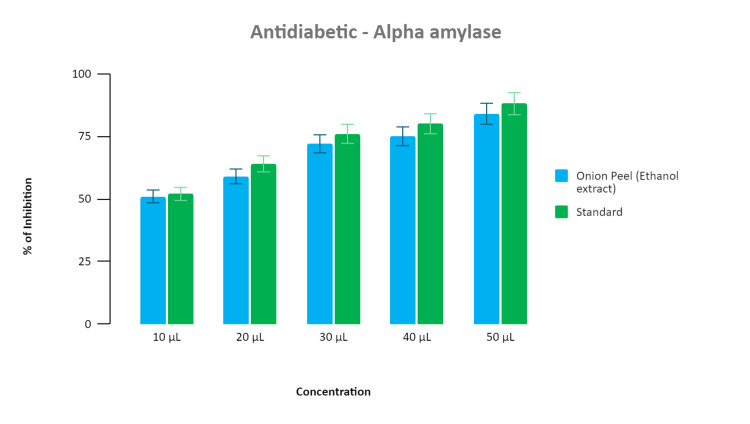
The graph illustrates the relationship between concentration of ethanolic extract from dried onion peel and inhibition of alpha-amylase enzyme. Our extract exhibits comparable inhibition to the standard diabetic drug, acarbose, with percentages reaching 50% at 10 µL, 75% at 30 µL, and 80% at 50 µL.

Remarkably, our findings revealed a significant similarity in the percentage inhibition of alpha-amylase between our extract and the standard drug. Beginning at a concentration of 10 microliters, we observed a 50% inhibition of the enzyme activity. This inhibition further increased with escalating concentrations. At 30 microliters, both the extract and the standard drug exhibited a substantial inhibition of 75%, highlighting the potency of our extract. Notably, at a concentration of 50 microliters, both the extract and the standard drug demonstrated an impressive 80% inhibition of alpha-amylase activity. These results underscore the potential of the ethanolic extract from dried onion peel as a promising therapeutic agent for managing diabetes (Figure 2).

Alpha-glucosidase activity

**Figure 3:**
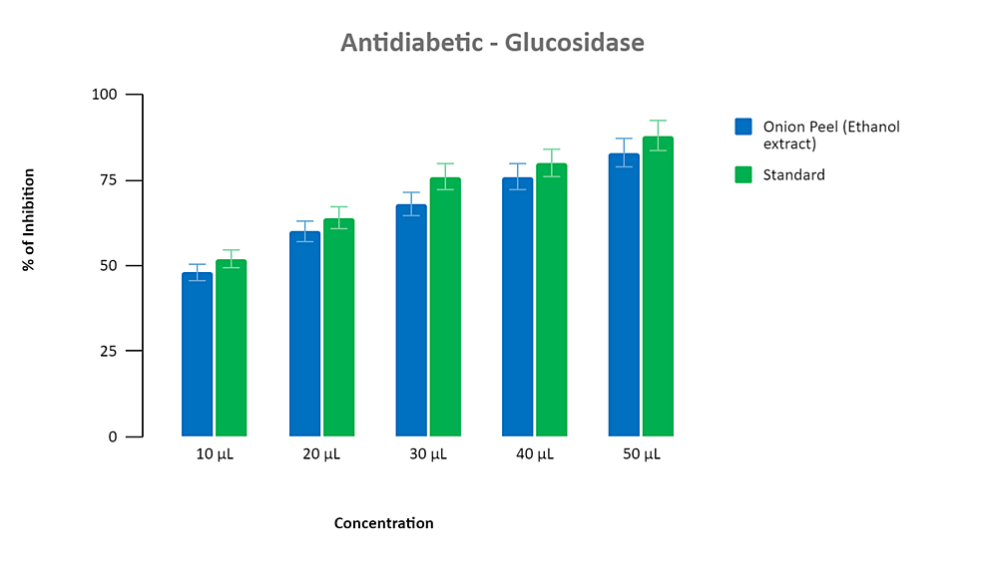
The graph illustrates the concentration-dependent inhibition of alpha-glucosidase enzyme by our extract compared to a standard diabetic drug. Both showed comparable inhibition percentages, mirroring the trend observed for alpha-amylase inhibition. This consistency underscores the effectiveness of our extract in managing diabetes.

The ethanolic extracts derived from dried onion peel demonstrate significant inhibition of alpha-glucosidase activity, leading to a reduction in blood glucose levels and an increase in insulin production. Our comparative analysis revealed a noteworthy similarity in the percentage inhibition of alpha-glucosidase between our extract and a standard diabetic drug. Concentration-dependent effects were observed, mirroring the trend observed for alpha-amylase inhibition. This consistency underscores the potential of our extract as an effective therapeutic agent for managing diabetes (Figure 3).

## Discussion

Rather than raising insulin levels and producing additional pancreatic effects, some articles [19] report or indicate that Allium cepa acts immediately as a hypoglycemic agent by acting on human tissues like the liver and muscles and changing or lowering the activities of the regulatory enzymatic processes of glycolysis, gluconeogenesis, and other pathways [20,21].

Kim et al. in 2013 stated that taking onion peel extract as a supplement caused a considerable drop in blood sugar levels by reducing the rate of glucose absorption by inhibiting the sucrase enzyme in the digestive system [23]. A number of processes lead to the absorption of glucose, beginning with the binding of insulin to cell surface receptors and continuing with the ability of insulin to boost glucose transporter (GLUT)'s transport of glucose inside muscle tissue [24]. 

The enzyme alpha-glucosidase, localized within the villi of the small intestine, plays a pivotal role in hydrolyzing disaccharides and oligosaccharides into D-glucose units, thereby facilitating glucose absorption. Inhibiting this enzyme activity can mitigate the rate of glucose absorption, consequently reducing postprandial plasma glucose levels [25]. Notably, research on phenolic-rich botanical sources has revealed a correlation between the antioxidant properties of extracts and their ability to inhibit alpha-glucosidase activity [26]. Among these botanicals, Allium cepa bulb has garnered attention for its diverse physiological effects, including its potential in diabetes prevention. Despite harboring substantial quantities of phytochemicals, studies investigating the anti-diabetic properties of onion peels and outer skin remain limited. Thus, exploring the therapeutic potential of these overlooked components could unveil novel avenues for managing diabetes.

Prior research has shown that quercetin exerts a mixed type of non-competitive inhibition, binding to both free enzyme and enzyme-substrate complexes. Its 3-OH glycosylated compounds can transition from passive to active inhibition. Chemical modifications, such as substituting the third hydroxyl group on the C-ring and adding hydroxyl groups on the B-ring of C3(A)-C6(C)-C3(B), enhance its alpha-glucosidase inhibition potential [27]. Quercetin's abundance in onion peels compared to bulbs may contribute to observed outcomes. Onion peel extracts demonstrate potent alpha-glucosidase inhibition, with phenol concentration likely influencing enzyme inhibition capacity. Overall, quercetin's mechanism of action involves inhibiting alpha-glucosidase through mixed non-competitive inhibition and chemical modifications, alongside enhancing insulin sensitivity, reducing inflammation and oxidative stress, and regulating gene expression, collectively contributing to its anti-diabetic activity [28].

Therefore, it is thought that the phytochemicals found in onions may improve glucose metabolism and insulin sensitivity by changing the activity of the insulin receptors and glucose transporters. Even smaller concentrations of onion peel extract were found to have a stronger effect than the comparatively greater amount of onion bulb powder [29]. As compared to the interior bulb, previous investigations have shown that onion peel has a rather high in vitro antioxidant capacity. The outer dry scales and fleshy peels of onions are significantly more abundant in phytochemicals like quercetin, isorhamnetin, and kaempferol than the inner layers [30]. Our study reaffirms onion's potential as an anti-diabetic agent, consistent with prior research. Through both in vitro and in vivo investigations, we observed onion's efficacy in inhibiting alpha-glucosidase activity, a crucial enzyme in glucose metabolism. Animal models treated with onion extracts displayed improvements in glucose tolerance, reduced insulin resistance, and enhanced pancreatic β-cell function, mirroring findings from previous studies. These results underscore onion's role in diabetes management, bridging the gap between laboratory research and potential clinical applications.

## Conclusions

The research demonstrates that the anti-diabetic effectiveness increases with ethanolic extract of dried onion peel concentration. In particular, the total phenolics, total flavonoids, quercetin, and its derivatives, which are contained in onion peel and skin, are highlighted in this study along with their therapeutic uses as a cardioprotective, anti-obesity, anti-diabetic, anti-cancer, and antimicrobial agent. The results of the current investigation demonstrate that Allium cepa’s ethanolic leaf extract has hypoglycemic and anti-diabetic properties. The presence of ethanol in the extract is responsible for its hypoglycaemic action. These substances are thought to be responsible for many plants' anti-diabetic properties.
